# A qualitative study of organisational resilience in care homes in Scotland

**DOI:** 10.1371/journal.pone.0279376

**Published:** 2022-12-20

**Authors:** Alastair Ross, Janet E. Anderson, Santhani Selveindran, Tamsin MacBride, Paul Bowie, Andrea Sherriff, Linda Young, Evie Fioratou, Edel Roddy, Heather Edwards, Belinda Dewar, Lorna M. Macpherson

**Affiliations:** 1 Glasgow Dental School, School of Medicine, Dentistry & Nursing, University of Glasgow, Glasgow, United Kingdom; 2 Department of Anaesthesiology and Perioperative Medicine, Monash University, Monash, Australia; 3 Cambridge Institute of Public Health, University of Cambridge, Cambridge, United Kingdom; 4 School of Health and Life Sciences, University of the West of Scotland, Glasgow, United Kingdom; 5 NHS Education for Scotland, Inverness, United Kingdom; 6 Centre for Undergraduate Medicine, School of Medicine, University of Dundee, Dundee, United Kingdom; 7 The Care Inspectorate, Dundee, Scotland, United Kingdom; Universita degli Studi di Milano, ITALY

## Abstract

Providing care for the dependent older person is complex and there have been persistent concerns about care quality as well as a growing recognition of the need for systems approaches to improvement. The I-SCOPE (Improving Systems of Care for the Older person) project employed Resilient Healthcare (RHC) theory and the CARE (Concepts for Applying Resilience) Model to study how care organisations adapt to complexity in everyday work, with the aim of exploring how to support resilient performance. The project was an in-depth qualitative study across multiple sites over 24 months. There were: 68 hours of non-participant observation, shadowing care staff at work and starting broad before narrowing to observe care domains of interest; n = 33 recorded one-to-one interviews (32 care staff and one senior inspector); three focus groups (n = 19; two with inspectors and one multi-disciplinary group); and five round table discussions on emergent results at a final project workshop (n = 31). All interviews and discussion groups were recorded and transcribed verbatim. Resident and family interviews (n = 8) were facilitated through use of emotional touchpoints. Analysis using QSR NVivo 12.0 focused on a) capturing everyday work in terms of the interplay between demand and capacity, adaptations and intended and unintended outcomes and b) a higher-level thematic description (care planning and use of information; coordination of everyday care activity; providing person-centred care) which gives an overview of resilient performance and how it might be enhanced. This gives important new insight for improvement. Conclusions are that resilience can be supported through more efficient use of information, supporting flexible adaptation, coordination across care domains, design of the physical environment, and family involvement based on realistic conversations about quality of life.

## Introduction

### Caring for older adults in care homes

Providing care for the dependent older person is vital and rewarding but presents many organisational challenges. Staff look after vulnerable residents with complex needs while balancing efficiency demands, resource constraints and policy and regulatory requirements [1]. Despite improvement efforts there have been persistent concerns about the quality of care in areas such as nutrition [2], oral care [3,4] pressure ulcers [5], and risks associated with polypharmacy [6] as well as a growing recognition of the need for holistic, person-centred care focused on quality of life [7].

Complex care and support needs underpinned by disability and comorbidity are placing increasing demands on staff [8] that have been exacerbated by the COVID-19 pandemic and response [9]. UK data suggest an over 200% rise in all-cause mortality in nursing homes [10] with dementia being the most common pre-existing condition for those whose death was recorded as COVID-19 [11]. In Scotland, around a third of COVID-19 deaths have occurred in care homes [12], and larger homes were more at risk [13].

The historical separation of health and social care has had detrimental effects on the staffing and funding of social care [14]. Programmes to improve safety and quality, which are now relatively mature in acute settings, are not implemented to the same extent in the social care sector, where important distinctions are made between home and institution, and between residents and patients [15]. Improvement efforts are primarily concerned with increasing staff knowledge, awareness and skills though education and/or training [16,17]. However, in complex care settings, interventions that do not consider broad system interactions between organisational processes and tasks, design and technology, and patient needs “are unlikely to have significant, sustainable impact” on safety and quality [18].

Across the wider healthcare landscape, the use of organisational theory and tools is endorsed [19] and the need to study healthcare as a complex system prior to designing and implementing improvement efforts has recently been formally recognised [20]. Traditional improvement strategies focus on identifying problems, for example from complaints, incident reports or inspection reports, and targeting their reduction. This retroactive, deficit-based endeavour has been termed the ‘find and fix’ approach [21] and its limitations have led to the complementary study of how success is achieved under difficult conditions as a basis for identifying improvements [22].

To address this gap in the application of healthcare systems theory to care home improvement, the aim of the I-SCOPE (Improving Systems of Care for the Older person) project was to systematically examine how care home systems deliver care, with a focus on how staff achieve positive outcomes by balancing multiple competing priorities in the context of dynamic conditions, to inform improvement efforts.

### Resilient healthcare

I-SCOPE utilised the systems-theoretical model of Resilient Healthcare (RHC). The international RHC literature presents an innovative set of ideas about how safe, quality care emerges and can be enhanced by focusing on how to support organisations in achieving outcomes which emerge from complexity in everyday work [23]. The idea of ‘resilience’ in RHC is concerned with “the capacity to adapt to challenges and changes at different system levels, to maintain high quality care” [24].

We applied the CARE (Concepts for Applying Resilience Engineering) model [25,26] which drove the research questions, observation guides, interview schedules and data analysis.

The model was developed to guide in depth fieldwork to identify organisational resilience and how it might be increased [26]. It is a generalised abstraction identifying the key theoretical concepts and the relationships between them so that they might be investigated empirically. Specifically, the model presupposes that full alignment between work demands and organisational capacity is rarely achieved, thus the care system has to dynamically adapt and adjust to achieve its aims. The distinction is thus made between what is intended or ideal (‘work-as-imagined’) and the actuality of everyday work (‘work-as-done’). To understand work as it is done in practice there is a need to study how dynamic adaptations occur. The CARE model has been applied in various hospital settings and has been extended to include factors that stimulate adaptation, and processes through which adaptations occur [27,28].

This theory-based approach is intended to be used to describe everyday work, which then allows for the identification of improvement opportunities. This is approached through a conceptual lens that examines five key organisational activities or potentials which support this everyday work, that is which are needed for a work system to function in a resilient manner: anticipating issues that may arise; responding to conditions and indicators; monitoring the system e.g. in terms of needs or outcomes; and learning from experience, e.g. what worked well and what could be done differently; coordinating tasks and resources [29,30].

### Aims

The overarching aims were: a) to systematically study and describe how everyday care is delivered and how staff adjust and adapt to achieve successful outcomes; b) to identify general areas for informing potential improvement in organisational resilience.

## Methodology

A qualitative methodology was chosen as most appropriate to meet the aim of describing how everyday work is performed and how staff, teams and organisations adapt to complex conditions and demands.

### Recruitment

Care Homes were recruited under principles of theoretical sampling [31]. A frame was built to ensure a spread across: type of provider; size of home; size of provider; geographical Health Board; residential or residential plus nursing homes. The ENRICH (Enabling Research in Care Homes) network, the Care Inspectorate and Scottish Care assisted with distribution of recruitment material by email outlining the aims of the research team.

All individual participants (staff in homes, focus groups, and workshop participants) were purposively recruited and formed a non-probabilistic sample to ensure sufficient spread to inform the study [32].

### Procedures

Observations, staff interviews, Focus Groups and round table discussions during the project Workshop focused on: what demands different protocols place on staff; how they reconcile competing demands and objectives within the context of administrative, resource, staffing and financial pressures; how care is co-ordinated under variable organisational conditions; what trade-offs and priority decisions are made and how; how technical and physical resources impact on care; and what opportunities exist for designing/supporting successful interventions in an integrated way, so that they do not lead to unintended consequences.

#### Non-participant observation

After agreement from the liaison group (see oversight), all homes facilitated broad, open-ended non-participant observation of care activity (shadowing staff) in the first instance. A field notes template [33] was drawn up to capture: time and place; descriptions of activities; people and materials involved; goals and reflections; emerging questions and potential areas for future study. Staff were asked questions discretely where possible. Subsequent observations were more focused and selective (see Table 1) based on emerging themes from initial shadowing of staff, and liaison group input on priority areas of care.

**Table 1:** Participating care homes and sources of data.

Source	Region	Sector/ Setting	Data gathered	Subtotals (Interviewer/ facilitator initials)
**Home 1**	Greater Glasgow and Clyde	Large private provider; residential and nursing care	Introductory visit; relatives meeting 1 hr; broad observation 6 hrs; targeted observation on medication round 3 hrs In-depth interviews: manager; senior carer; nutrition champion; nursing assistant (2)	Observation 10 hrs Interviews n = 5 (SS, AR)
**Home 2**	Greater Glasgow and Clyde	Small private provider; residential and nursing care	Introductory visit; broad observation 7 hrs; targeted observation on nursing care 6hrs In-depth interviews: manager; senior carer; dietician; carer (2)	Observation 13 hrs Interviews n = 5 (SS)
**Home 3**	Tayside	Small third sector provider, residential care	Introductory visit; relatives meeting 1 hr; broad observation 7 hrs; targeted observation shadowing team leader 4 hrs; social activity observation 3 hrs In-depth interviews: manager; senior carer (2); activities coordinator; carer	Observation 15 hrs Interviews n = 5 (SS)
**Home 4**	Lothian	Small third sector provider, residential care	Broad observation 7 hrs; targeted observation on care plan review 1 hr; targeted observation overnight 8 hrs In-depth interviews: deputy manager; senior carer; carer (2)	Observation 16 hrs Interviews n = 4 (SS, AR)
**Home 5**	Greater Glasgow and Clyde	Local Authority provider, residential care	Introductory visit; broad observation 5 hrs; targeted observation on medication round and oral care 3 hrs In-depth interviews: manager; social care assistant; social care worker (3)	Observation 8 hrs Interviews n = 5 (SS)
**Home 6**	Lothian	Small third sector provider, residential care (sister home to home 4)	Broad observation (social activity; mealtime; medication round) 6 hrs	Observation 6 hrs
**Homes 7–11**	West of Scotland	Linked to My Home Life Programme; 3 residential and nursing care; 2 residential care	Training workshop with staff; Short interviews or dyadic interviews: residents (3); relatives (5)	Interviews n = 8 (SS)
**Interview**	National	Care Inspection	In-depth interview: Senior Care Inspector	Interviews n = 1(SS)
**Focus groups**	National	Care Inspection	2 x Focus Group discussions: Care Inspectors and Team Managers/ Improvement advisors	Focus group n = 11 (SS)
**Focus group**	West of Scotland	Multi-disciplinary	Focus Group discussion: Care Home Manager; Dentist; Oral Health Co-ordinator; Dietician; Physiotherapist; Pharmacist)	Focus group n = 6 (AR)
**Workshop**	National	Focus on oral health; then resilience themes	Round table discussion: care staff, oral health educators, dental public health professionals	Round table discussion: n = 8
**Workshop**	National	Focus on medication; then resilience themes	Round table discussion: care staff, staff nurse, nursing assistant, GP, pharmacist	Round table discussion: n = 7 (JA)
**Workshop**	National	Focus on social activity; then resilience themes	Round table discussion: care staff; activities coordinator; software designer	Round table discussion: n = 6 (SS)
**Workshop**	National	Focus on nutrition; then resilience themes	Round table discussion: care staff; nutrition coordinator, nursing academic	Round table discussion: n = 6 (PB)
**Workshop**	National	Focus on mobility; then resilience themes	Round table discussion: care staff; software designer; care sector representative	Round table discussion: n = 6 (AR)
**Totals n = 75** **8 care home managers; 7 senior carers/ deputy managers; 12 carers/ nursing assistants; 1 nutrition champion; 2 dieticians; 1 activities coordinator; 3 residents; 5 family members; 1 senior care inspector; 12 care inspectors/ improvement advisors; 3 dentists; 1 oral health coordinator; 1 physiotherapist; 2 pharmacists; 3 nursing professionals; 2 General Medical Practitioners; 4 Public Health Professionals; 3 care directors; 1 care software engineer; 1 senior social scientist; 2 industry representatives**				Observation hours 68 Interviews n = 33 Focus Groups n = 17 Round table discussions n = 33 (incl. 8 previously interviewed)

#### Staff interviews

Through discussion with managers, carers and senior carers were recruited purposively to inform the aims of the research and to ensure a range of perspectives were gathered. All staff interviews took place one-to-one in care homes using private rooms. Interviews were piloted with two care home managers; these were included in analysis with consent. This involved broad questions to start, then targeted questions and prompts with regards to personal care, medication, mobility/ falls risk, nutrition/ weight management, oral care, and social activity. This structured approach did not preclude the emergence of data themes which apply across all these areas, such as frailty and end of life care, dementia care, holistic outcomes such as quality of life, and general functions such as managing staff, care planning and administration.

#### Focus groups

Three Focus Group discussions were held in central locations with travel remunerated: two with staff from the Care Inspectorate including Team Managers and Senior Improvement advisors (recruitment facilitated by HE); one with a multi-disciplinary group from liaison teams providing a focus for multi-disciplinary working across homes (recruitment purposive facilitated by care home staff).

#### Resident and family interviews

In initial discussions, our advisory group suggested that involving residents might be best approached by briefing care home staff to conduct short interviews. It was felt this would be less invasive and have less potential for distress than if residents were approached directly by members of the research team.

We collaborated with My Home Life (MHL) to recruit residents and family members and to gain expert advice on conducting the interviews. MHL is an international initiative that aims to promote quality of life for those living, dying, visiting and working in care homes. It is underpinned by relationship centred care [34], Appreciative Inquiry [35] and Caring Conversations [36] with a focus on developing best practice collaboratively. After ethical amendment to allow the involvement of care home staff in fieldwork, we held a collaborative briefing event with staff already exploring care by engaging with residents through their participation in the MHL Leadership Support and Community Development programme [37]. We designed resident and family member interviews/dyads to be carried out by staff using ‘emotional touchpoints’ [38], an approach to interviewing that enabled us to explore experiences relevant to our research in a structured way focused on emotions. The briefing allowed us to outline our research questions and aims and ensure they could be explored using this modality. Organisational principles from the new Health and Social Care Standards [39] were used as a guide to elements of daily care that were then introduced in a neutral manner. These principles outline care expectations e.g. “I have confidence in the organisation providing my care and support”. Participants were asked to select from a range of words (for example: comfortable; encouraged) that summed up how they experienced these, and prompted to explain why. This approach was selected as it can enable individuals to describe feelings in a way that makes sense to them and captures aspects of care that can be hard to define and/or incorporate in standard interview schedules [40].

#### Project workshop and round table discussions

A final project Workshop was held, also in a central location. This brought together researchers, care home participants and wider professional stakeholders (including those from policy, regulatory inspection, medical, nursing, dental, pharmacy, nutrition and psychology backgrounds) to discuss findings and recommendations. We chose the method to work dually as an engagement event to maximise impact of the research and as a chance to conduct further in-depth discussions focused on the main emergent findings/ themes. Recruitment as above was purposive through contacts at Scottish Care, the project liaison group (care home managers) and the advisory group.

After talks from project researchers and managers and carers reflecting on the work, five round table discussion groups were facilitated and recorded. Topics were chosen based on thematic findings from the fieldwork. Excerpts (either direct quotes or notes from observation) were presented as prompts for separate discussions on oral health; medication; social activity; diet and nutrition, and falls/ mobility. Participants were told these were initial themes rather than definitive findings, in order to generate reflection and debate. Then all five groups were given prompts for a more general discussion on the emergent themes in terms of organisational resilience, and again asked to give their opinions and reflect on any implications.

### Analysis

QSR NVivo 12.0 was used to manage transcripts, notes, images and care documents. All interview, Focus Group and Workshop Round Table discussions were digitally recorded and transcribed verbatim. Audio recordings and photographic images were frequently used to supplement observations and facilitate note taking. Interview notes were also taken and collated. In addition, interviews with staff often involved discussions of care documents and artefacts such as care plans and notes, electronic monitoring systems, resident charts, inspection documents, and protocols and guidance. The number of interviews and focus groups was finalised based on principles of data saturation, where no new themes were deemed to be emerging from analysis [41].

A two-stage analysis was carried out in line with RHC methods and guidelines for applying the CARE model. This theory-based approach is relatively deductive, in that RHC provides a frame within which to describe everyday work and examines this with a focus on resilient performance (or otherwise). Two experienced coders of RHC data and the main project researcher (AR, JA, SS) analysed and coded excerpts using the CARE model concepts and the underpinning resilience activities [42]:

- **Capturing work-as-done.** First, contextual examples from different areas of care were extracted, describing everyday work or ‘work-as-done’ in terms of the interplay between demand and capacity, adaptations (e.g. trade-offs and priority decisions), and intended and unintended outcomes. This gives a rich description of how outcomes emerge from the interplay between complex system conditions and dynamic adaptation/ adjustment (Table 2).

**Table 2:** Examples of ‘work-as-done’ in care homes, viewed through the CARE resilience model.

General context/ care domains	Short description	Illustration [source]	CARE coding of work-as-done
End of life care	Prioritising end of life care in the context of providing for all residents	[…] *staffing levels*, *you know*, *that could put a wee bit of strain on you as well*. *[…] like you’ve got somebody end of life but you’re trying to prioritise that over other things and you’re struggling* […] *because you’ve got so many people to look after and to give them the best that you possibly can* […] *basically*, *it’s trying to time manage and make sure that you’re alright and you’re getting priorities elsewhere* […] *if you’re short-staffed*, *you just need to put things into priority*, *your residents come first obviously over any paperwork*, *then you get to wee jobs*. [Senior Carer, Home 1, Interview]	Goals: Optimal care for all residents; prioritising end of life care Demand/capacity considerations: Short staffing; time pressure; paperwork and other tasks Adaptation/adjustment: Prioritise residents/ direct care over paperwork Impact/ outcomes: Residents get best care; paperwork may suffer; delay in carrying out lower priority tasks
Mobility	Assessing residents’ dynamic mobility needs and involving them in decisions; being responsive	*Sometimes somebody might be using a stand aid*, *but they might not need it every single time*, *depending on how they are*. *But that again takes a wee bit of experience and* [knowing] *that resident* […] *Rather than just automatically going*, *‘this person was assessed’- they can use a stand aid*, *so we’re going to use it’*. *It’s actually*: *how do you feel today*, *so-and-so*? *what do you think*? *And you’re involving the resident in that decision*. *So I think it’s about having these discussions with the resident as well and it’s not prescriptive*. [Carer, Home 4, Workshop discussion on mobility]	Goals: Optimal use of mobility aid; involving residents in decisions Demand/capacity considerations: Residents’ dynamic needs; assessment can be prescriptive; staff experience needed Adaptation/adjustment: Asking residents how they feel; what they think Impact/ outcomes: Stand aid use might be at odds with current mobility assessment
Medication	Working collaboratively to deliver timely medication	*If you’ve got two units of meds to do and then you’ve got staff that need to cover breaks* […] *say for instance* [resident A], *she’s not woken up ‘til maybe half nine*, *we’ll know we don’t need to rush with her*, *so we’ll maybe try and do* [resident B] *first*, *or if* [resident B]*’s not got up ‘til later*, *we’ll do* [resident A] *first*, *so we kind of work it like that*. […] *For instance*, *I know when I’ve done my medication* […] *if it’s pain relief*, *they can’t get that ‘til half 12*, *so what we’ll do is I’ll say*, *well*, *I’m free for half an hour*, *so somebody can go on their break and I’ll feed* [resident], *so we kind of work it like that*. *I mean*, *it is good teamwork*. [Social Care Worker, Home 5, Interview]	Goals: Cover staff breaks; provide timely medication Demand/capacity considerations: Variability in time when residents may wake up Adaptation/adjustment: Change order of care for residents; change order of staff breaks Impact/ outcomes: Breaks covered and medication delivered in line with guidance
Weight management	Nursing staff oversee information for care planning to ensure weight loss will be picked up	*We would get the carers to weigh the residents and we found that* […] *a lot of them don’t understand the BMI and things like that* […] *So what we’ve decided now is that the carers take the weights and give myself*, *there are two nursing assistants or the nurse*, *the weights and we’ll put it into the care plan*, *that way I don’t miss anything* […] *If I had somebody’s MUST [Malnutrition Universal Screening Tool score] at two*, *I was coming back and it was a one*. *Do you know what I mean*? […] *So I just felt*, *no*, *we need to keep on top of it a bit better than that*. *It wasn’t their fault* […] *but they’ve not realised in the last six months they’ve lost the 10% so they weren’t changing the score*. [Nursing Assistant, Home 1, Interview]	Goals: Monitor weight accurately Demand/capacity considerations: Carers not understanding the metrics used Adaptation/adjustment: Carers record weight and pass to nursing assistant or nurse to update in care plan Impact/ outcomes: Better monitoring
Oral care	Maintaining good oral hygiene through flexibility e.g. adoption of palliative adaptations	*We’ve got a resident in here who won’t let us brush her teeth at all*, *so it’s really quite hard trying to get the toothbrush into her mouth*, *but there’s other ways you can do it*, *like try with a wee bit of gauze and things like that and try and clean her mouth*. […] *We’ve only been trained to do that in palliative care but we just do it sometimes*, *like if she’s stored food in her mouth or things like that but* [name] *is quite hard to deal with because […] she’ll scream and she’ll shout*, *so you’ve got to just*, *kind of*, *play it by ear because she might not do it in the morning but she might let you brush her teeth in the afternoon*. […] [name] *has also got mouthwash but it’s normal*, *it’s not prescribed […] We’ve got to*, *kind of*, *watch because then she’ll try and just drink it*. *So you’ve got to watch that as well with dementia*. [Social Care Assistant, Home 5, Interview]	Goals: maintain good oral hygiene with clean teeth, dentures, mouth Demand/capacity considerations: residents with dementia may resist brushing; risk re. ingestion Adaptation/adjustment: using wetted gauze on finger; being flexible with times; watch when using mouthwash Impact/ outcomes: prevent infections; maintain dignity
Nutrition	Champions can provide information when dietician input is delayed	*I think it can work in some cases* [where] *they’ve got nutrition champions and they run the nutrition champion programme*. *[…] As a dietitian*, *we’ve got such a high clinical case load that we can’t be in the care homes daily or weekly; it can be up to six weeks before we’re back in that care home again*. *So it’s a port of call for the staff that they can go to*, *somebody with a little bit more expertise*, *and I think it helps that member of staff as well–maybe they feel more purpose as well*, *they’ve got that knowledge and that information*. [Community dietician, multi-disciplinary Focus Group]	Goals: give nutrition advice Demand/capacity considerations: high clinical case load for dieticians Adaptation/adjustment: using staff trained to be ‘champions’ Impact/ outcomes: distributes expertise and empowers the champion themselves
Frailty	Manage expectations with increasingly frail residents	[…] *some people came in 10 years ago*, *15 years ago*, *because they were bored*, *and they were alone*, *or whatever*, *you know*. *Because Care at Home didn’t really exist in these days*. *But now*, *by the time they come in*, *certainly by the time they come into a nursing home*, *they’re very*, *very frail* […] *I mean*, *again*, *five years ago*, *they would have been going into hospital*, *but they’re now coming into a care home at that point*. *So*, *you know*, *some relatives’ expectations are that at some stage their mum*, *or dad*, *or auntie*, *or uncle*, *will perhaps go back home*, *or walk out*, *or you know*. *So it can be quite trying*, *sometimes*, *trying to*, *you know*, *balance up their expectations*, *with the resources we have*, *and the care we do*. […] *I mean*, *you know*, *one example would be*, *someone who is in a semi-conscious state*, *PEG fed* [percutaneous endoscopic gastrostomy, feeding tube], *you know*, *and yet they still want them to be resuscitated*, *you know*. […] *I think that*, *then*, *takes leadership*, *you need to have your nurse there to speak to the family*, *and tell them what the realistic expectations are*. *And any nurse worth their salt would do that*. [Care provider/owner, Workshop discussion on medication]	Goal: manage frailty Demand/capacity challenges: increasing frailty; relatives expectations can be unrealistic Adaptation/adjustment: explaining Impact/ outcomes: balance expectations with resources and resident needs
Falls risk	Adapting to falls risk/ limitations in the physical/ built environment	*We try and minimise the risk to a level that they need to maintain their independence […] we can have up to 150 falls a month*, *depending on people’s mobility*. *And that’s why we had to design the rooms specifically and the corridors specifically so that if people did fall*, *they wouldn’t hurt themselves*. *The corridors have been made bigger […] to allow people to take the risks*. *Everything’s coloured coded to a certain level that they recognise where the bannisters are so they can use that to maintain independence*. [Care home manager, Workshop discussion on mobility]	Goal: manage falls risk and optimise mobility Demand/capacity challenges: older homes with stairs Adaptation/adjustment: design of corridors Impact/ outcomes: balance safety/ risk with independence

- **Describing resilience in everyday work.** The next step was to synthesise data from step 1 into a higher-level thematic description which gives an overview of resilient performance. This involves examining the breadth of ‘everyday work’ (Table 2) to identify where the five resilience activities are indicated as supportive (or not), that is: where the work system more generally is functioning in a resilient manner or otherwise and thus where attention for intervention can be focused. Table 3 shows three main themes that emerge from this stage of analysis. These cut across the different domains of care and thus characterise organisational resilience whereby “the output from this step should be a comprehensive overview or map of the resilience in the system” [29].

**Table 3:** Themes in everyday work, with description of key resilience activities.

Themed area	Descriptions of resilience activities	Illustrations
**Care planning and use of information**	Monitoring / anticipating is hindered by cumbersome documents without a clear focus; streamlining is felt to be beneficial alongside supporting staff capacity to develop appropriate plans	“People are often complaining about the amount of paperwork that they’re doing […] I think, often, it is just straightforward, pure, or cumbersome systems that they are using. […] From my point of view, what we often see is that care plans are not clear about the focus that they are supposed to have. So you’re storing yourself up problems in the long run […] trying to compensate for the lack of quality with quantity […]” [Witheld]. […] the other thing is, each company [uses] different assessment tools or dependency tools to gather the data. So I think there should be a streamlined dependency tool across the care home sector that we utilise rather than individual ones. [Care Home Manager, Workshop discussion on mobility] […] sometimes, we have a workshop on how to develop a care plan […] we have an input. So now, that goes for every care plan that we have written, whether it’s for the oral, whether it’s for the nutrition, whether it’s for the moving and handling […] [Manager, Home 1, Interview]
	Responding to issues can lead to care plans being deprioritised; learning often comes from other homes; electronic systems can improve monitoring through efficient use of information	[…] It’s good for us when you get a good care plan, but it’s very difficult to manage when you would do direct care for people, vulnerable people. And it’s the time factors that that takes. That takes a lot of time, collating all this information […] it’s a vital tool that we need to have in place [but] they’ll say that that’s the first thing that will go, is the care plan if something happens, if they’re having to support and assist people. [Manager, Home 5, Interview] Care managers will look around and pick up evidence from different places and if there’s a good idea, will put it forward to, to our team and see what we think, and maybe test it out [Manager, Home 2, Interview] With [e-system] I can look at any resident within [provider name’s] care plan and I can look at their profile and everything so that my treatment matches in with what else is happening elsewhere. With a paper record you were opening it up and looking, you know…’what did they do yesterday, what did they do today’, all that kind of thing. So I think electronic records have a huge potential to improve quality of care. [Head physiotherapist, multi-disciplinary Focus Group discussion]
	The design of buildings can hinder or help staff when planning care and responding to, for example, dementia and mobility needs	“But care homes have evolved throughout the years, [previously] nine out of ten times an older person, their mobility was reduced. So it wasn’t designed for that need. Whereas now older people are living longer even though their needs have changed, but homes have never always been adapted to work that way. [Manager, Home 1, Workshop discussion on mobility] […] certainly in our place […] I think there are, sort of, positives to do with the physicality of the building, ‘cause as soon as you’ve got a set of stairs, then you’re basically saying that for some residents that want to go upstairs/downstairs/go out to the garden, they’re going to become more reliant obviously on a member of staff to facilitate that. So [,,,] that actually does reduce someone’s personal mobility when, if you want to enjoy some fresh air, you have to, kind of, request it. [Carer, Home 4, Workshop discussion on mobility] It’s a nice big space but it’s not very dementia friendly. […] A lot is still about the kind of architectural issues and garden issues. I mean, it is a big area. […] you don’t want them to be unsupervised […] that’s why the staff are very good at bringing people out for walks. [Activities coordinator, Home 4, Interview]
**Coordination of everyday care activity**	Senior staff are vital in responding to conditions and coordinating a wide range of activity; staff meetings monitor how to make things work	[…] we’re usually in the duty room by 7:15 and we get the night report. […] it depends which part you’re working in. We knock on their doors. Good morning to them all. We do that to start with. And then we go back and if any are needing help, we support them. […] Some of them go along to the dining room, so they may need support along to the dining room, they may not. […] it’s a case of supporting ones that need help with personal care, oral hygiene, continence, making sure that their room’s tidy, dirty glasses are taken away. […] So, by the time you get round to all of that it could be breaks. […] If there’s any activities going on that morning, which there sometimes are in the lounge… If anybody needs supported along there by wheelchair or walking or whatever, we take them along. [Care Worker, Home 3, Interview] That’s when you sit down and you have your regular staff meetings. You communicate. You have your handover meetings, you have…we have falls teams meetings, we have health and safety meetings, we have meetings with my trained staff and my nursing assistants. I have general staff meetings with the rest of the staff. And this is what we discuss. Things like that. If something’s not working, we look at, well we can make it work, you know. [Care Home Manager, multi-disciplinary Focus Group discussion]
	Learning how to coordinate care is through experience rather than formal training	I mean, you know, sometimes you think, [training] it’s not relevant, a lot of it you think is common sense, you know? I mean, every day is a learning experience, you know, we don’t know everything, we learn something new every…even I learn, you know, you learn something new every day. You learn what works, and then you find out, is that safe to do that? [Senior Carer, Home 4, Interview] Aye, you just need to kind of play it by ear […] You get to know the residents and what they like and what they don’t like; what they’ll tolerate and what they’ll not tolerate. As I say, it’s all a learning process, so it is. It’s all a learning process for everybody. […] We’ve got a new resident coming in tomorrow so she’ll need to get to know us and we’ll need to get to know her; it’s all learning–you get to know the resident. [Senior Care Assistant, Home 2, Interview]
**Providing person-centred care**	Systems for bringing together different services and specialties are important for coordinated care, for example in integrating physical and mental health	[…] we link in with the care home liaison team which now has pharmacy support team that come in […] We’ve just got a CPN [Community Psychiatric Nurse] allocated to us from the care home liaison team–they’ve added mental health services for older people, […] I think it’s seven or eight homes they have and they’re dedicated to them so referrals go through them from the care home liaison team now as well. And they’ve got access to different services; dietetics, physio, Caring for Smiles [oral health], different things […] [Care Home Manager, multi-disciplinary Focus Group Discussion] I feel really strongly that there are barriers between physical and mental health care, and how we overcome that, I think needs to be a priority. I think it makes it difficult for care home staff, as well, when it’s, there’s pockets of speciality, if you like. I think there needs to be an opportunity to have a system in place where there’s representation from a lot of specialities at any given time. [Team Leader, Mental Health Liaison Officer, Workshop discussion on nutrition]
	Staff are mentored to monitor holistically	Some of the residents I have are only really able to manage fluids. So for those people, we’re ensuring that they’re getting good quality of life as much as possible, that we’re keeping them well hydrated, and that we’re giving them foods and drinks that they like […] And it’s about screening and monitoring somebody’s nutritional status. […] is it somebody that’s constantly got chest infections, is that a sign that they’re maybe aspirating, are they constantly getting UTIs, they’re not drinking. Have they got wounds, you know, pressure wounds, that’s a sign of, you know, poor nutrition […] there’s a whole host of things […] that’s what I’m trying to get across to staff […] it’s getting them to think about the holistic approach, it’s not just about the weight. It’s, you know, having a wee checklist in their mind–has this been happening, has that been happening […] [Dietician, Home 2, Interview]
	Family input is important so that staff can know residents and monitor/ anticipate when something is wrong	“Most of the staff are fine. It’s a hard job but as I say, occasionally, especially in the early days, I felt I was very much trying to introduce them to how my mum was. And I sometimes felt, you’re not listening to me. You’re not taking on board this wee lady has lived her own life and been very independent” […] [Relative, Home 8, Emotional Touchpoints conversation]. [Re. resident’s daughter] She knows all the ins and outs and she’s awfully good in here at talking to the people. Oh, yes. And so, that was it. Everything was made for me to come in here beside [name], my wife. And, well, it went quite well I must admit. [Resident, Home 9, Emotional Touchpoints conversation] I think the…well, the staff, they understand. They see if you’re unhappy and they’ll say, is there something wrong? […] They’re very good at [that] most of them, they’re very good that way. [Resident, Home 10, Emotional Touchpoints conversation]
	Inspections that focus on residents rather than paperwork align with intended outcomes; provider monitoring of quality should align with regulators and commissioners	That’s what I liked about this new inspection we had this time- it didn’t focus on paperwork. She came in, she had a short talk with me and she then went out, wandered around the home, then came back with three or four names of residents, look at their care plan, then went out and spoke to my staff. And they could tell exactly what the key to that person was. […] she managed to speak to their relatives ‘cause they were in that few days visiting. And it was, like, a full circle. It was not paperwork…you know, whereas before it was all,’ can I see your health and safety, can I see your fire’…all that had gone. It was all about residents. One of my residents said to her…she’d asked, what do you feel best about? And she went, I feel safe. And she said, that was it. That’s all she wanted to hear, that the resident was cared for and felt safe and secure. [Manager, Home 1, Workshop discussion on mobility] I think the way the inspection is going, they lean, maybe more, as a visual inspection, that they’ll see what’s going on with their eyes, rather than read bits of paper, which could lead you down the wrong path quite easily. I don’t say that we get it right all the time, but we try our best. [Deputy Manager, Home 1, Workshop discussion on nutrition] […] the provider needs to look themselves, you know, that the people who are doing the internal compliance work, and the quality assurance work, are really well tuned in to the council, and their regulator. […] if they are starting to look inward too much and are trying to kind of do slightly different things, that’s when it becomes most confusing for the care managers, for the care home managers. Because then, you can come into that kind of situation where you find yourself being pulled in three different directions. [So] you need to be adaptable, and you need to be able to listen to your regulator, and to your commissioning team. And you need to kind of factor that in. There’s nothing wrong, if you want to be innovative, [but] if there is a provider who constantly feels that their own quality assurance programmes are not in tune with the regulator, or the commissioning team, then I think they should maybe look at that and see why that is. [Withheld]
	Expectations from families about responding to deterioration can be unrealistic; new health and social care standards set high expectations but are good to work towards	The family is a big thing. The expectation of something to be fixed, when there isn’t a solution, you know, […] and you maybe see quite a rapid deterioration, when you’re preparing, like, this is where we’re going with this decline, it’s not reversing itself. Which it can sometimes, if it’s a delirium, and you can treat the infection with antibiotics, extra fluids, what have you. But often, not. But the family’s expectation was, that last week they could walk, you know, last week they could eat by themselves; ‘last week, yesterday’, you know. I think there just comes, sometimes it is just from a day-to-day basis, that there’s a drastic change in somebody’s demeanour. And I think the family […] you know, and they’re not ready for where their journeys taking them. That it is maybe the beginning of end of life care […] [Deputy Manager, Home 1, Workshop discussion on nutrition] I think it could be read to really raise expectations which I think has probably always been scary. I mean, I can remember the Patients’ Charter going up in the hospitals and we’re all going, oh my God, don’t do that–no, no. So when I was reading through I was thinking, oh my God, that is a lot of expectations being set; whether they are things they work to or… I can’t imagine we would ever manage to achieve all of them with everybody; I think it’s just impossible. But I think, as guidelines to work towards, I think they’re good. [Activities coordinator, Home 4, Interview]

Coded excerpts describing work as done and resilience themes were put forward as propositions and tested during round table discussions with stakeholders at the project workshop (see Table 1). Stakeholders reviewed the analytic propositions and provided recorded comments and feedback which validated them as reflective of their own experiences and opinions on the characteristics of the work system.

### Reflexivity

The team involved registered nursing staff as well as psychologists and four qualified ergonomists. However, the principal research associate at the time was medically trained which may have influenced her observational note taking and aspects disclosed by staff. Residents were engaged by staff who knew them well. We believe this was the best course of action but prior assumption and experience may influence data gathered.

### Project oversight, ethical approval and consent to participate

An external scientific advisory group gave input at an early stage and a project liaison group was formed with managers of case study homes (there were no existing relationships between researchers and participants). Ethical advice was sought from the local NHS Research Ethics Service and the officer with special responsibility for adults with incapacity, who advised that under the terms of the governance arrangements for research ethics committees in the United Kingdom, NHS ethical review was not required and university approval would suffice. NHS approval was ultimately deemed unnecessary and thus the study was approved in writing by the University of Glasgow (College of Medical Veterinary and Life Sciences) Institutional Review Board (Project Ref: 200150178). Reporting follows COREQ guidelines [43]. All interview and Focus Group participants gave fully informed, written consent.

## Results

Table 1 shows data sources. It can be seen from Table 1 that there were 68 hours of observation during daily delivery of care, n = 33 one-to-one recorded interviews, and n = 50 respondents took part in Focus Group discussions.

### Capturing work-as-done

Everyday care for residents is characterised by staff adapting and adjusting to multiple dynamic demand and capacity issues to achieve positive outcomes. Table 2 shows illustrative examples, giving an indication of how this is widely manifest and characterises organisational activity across various domains of care.

Table 2 shows a complex care landscape. Residents’ needs fluctuate over time and are hugely variable in terms of frailty, cognitive capacity, personal choice, family involvement and other factors. Demand on the system, including in residential as well as nursing homes, is increasing and there are constant dynamic changes to conditions and variable interacting issues to deal with. Staffing can be an issue and time to provide care in line with residents’ needs and preferences is at a premium. All areas of care are supported by staff and organisations being flexible and adapting to circumstance.

### Describing resilience in everyday work

The next step in analysis was to induce higher level resilience themes which apply across the domains of care in Table 2 and are described below: Care planning and use of information; Coordination of everyday care activity; Providing person-centred care.

Table 3 contains further illustrative quotations, with short descriptions of how the key resilience activities (anticipating, responding, monitoring, learning and coordinating) are observed to be present, or to have room for improvement, under each of the three theme

#### Theme 1: Care planning and use of information

Information flow (verbal and non-verbal) is key to care monitoring and learning about what is working. Care plans and incorporated risk assessments work best when they are adaptable and used flexibly:

‘[care plans are] not going to be that rigid [because] a care plan is a live document’ […] this is what the patient is able to do, and this is what we’re able to do, and this is what we’re doing. Are we all happy with that? And is this benefitting our resident? Yes, or no? If it is not benefitting, how it’s not benefitting? Did we mess up something? […] Is the person comfortable there? Are they rightly placed there? Are they getting what they’re supposed to be getting? Are we doing the things the right way”?

[Deputy Manager, Home 4, interview]

However, monitoring can often be prone to inefficiency, which increases demand and erodes response capability. Documents can be seen by carers as primarily an administrative requirement and the ability/ motivation of care staff to document care through written process is highly variable. Managers share learning on care plans but documentation often proliferates in an ad hoc fashion and don’t seem clearly focused, increasing demand on staff and impacting directly on time spent with residents.

Some ‘streamlining’ of tools so that they are efficient monitoring and anticipatory tools is felt to be beneficial. In general, there are positive orientations towards electronic care monitoring and planning systems and their potential to reduce demand and improve ‘real time’ monitoring and anticipating. Finally, planning care and responding to need can be constrained by capacity in the built environment. Aspects such as stairs, gardens, size and function of units (e.g. palliative units) provide the context whereby care is carried out and again this requires staff to consider priorities, monitor and anticipate risk, and make adaptive decisions on an ongoing basis (Table 3).

#### Theme 2: Coordination of everyday care activity

Successfully caring for multiple residents with complex needs does not arise from simply following protocol, but from flexible responding. Success requires constant priority judgements, underpinned by coordination, anticipation, and response to emergent issues:

[…] when we come in, the night shift give us the handover […] let us know anything major happened, whether anybody’s fallen during the night or anything like that that we need to chase up during the shift. They also hand in the medication audits that they do every night. So we check those and obviously if there’s been any discrepancies […] we need to deal with that then. […] My next thing that I do is […] I would go round each of the units and do my temperatures. Anything that I need from either a member of staff or a resident while I’m in each unit I would attend to at that point. […] Then I’m looking at the diary to see if there’s any GPs or activities that are on that we need to accommodate. […] Throughout all this there’s visitors coming in that want to see you, there’s incidents that the staff need support, like today, with behaviour with residents. Like, families are popping in, handing in money, things like that.

[Senior Social Care Worker Home 5, interview]

Importantly, learning in this vital area is described as happening mainly through experience rather than through formal reviews or educational efforts. Local management/organisational culture is key to developing staff skills in prioritisation decisions and coordination of tasks and activities. At an organisational level, liaison teams facilitate coordinated care by bringing together different services and specialties.

#### Theme 3: Providing person-centred care

Treating residents as individuals and focusing on quality of life involves bringing together different aspects of care needs and co-ordinating services. Staff, residents and relatives broadly welcome the move towards person-centred and holistic models of care in line with the new health and social care standards in Scotland. Staff prioritise getting to know residents, anticipating what is needed accordingly, and attending to ‘the little things’ that enhance overall quality-of-life (though once more capacity in terms of staffing can be an issue):

What would I do tomorrow if things could be different? […] I think supporting the staff more on the floor; that’s a big thing we have to look at. […] You have to look at dependencies, and then that indicates how many staff we need for each resident. To improve someone’s quality of life […] it should be [about] what the staff are actually doing to make that person’s life better. It may be the slightest thing, but the staff don’t get the opportunity to do that sometimes. Every moment counts for a resident, that’s for sure, but it’s how that’s achieved, and sometimes there’s not enough staff to do the little things, the little quirks that the individual likes, listening to hymns or whatever; that or getting taken to the café somewhere one day a week or whatever–there’s just not sometimes the staff to be able to do that.

[Care Home Manager, multi-disciplinary Focus Group discussion]

Care planning and monitoring can still be somewhat compartmentalised into different domains. Resident and family input is vital, but family expectations can be unrealistic. As above in terms of coordination, care liaison teams that provide a platform for integrated services are discussed in positive terms (Table 3).

Assessment, inspection and evaluation need to be based firmly on realistic expectations of care outcomes and processes. In terms of monitoring outcomes, there is some concern about how to provide clear evidence for outcomes based on overall quality-of-life when it comes to inspection. Providers need to liaise with regulators and commissioners to align their own internal quality measures with external ones. Good inspection is described as being similarly holistic in focus, rather than focused on documentation of processes *per se* (see Table 3).

## Discussion

### Main findings and recommendations

This study aimed to examine complex work conditions in Care Homes. An in-depth qualitative study across multiple sites was conducted over 24 months drawing from ethnographic principles which are common in the RHC field [44] due to the need to gain a rich understanding of everyday work [45]. The study generated a comprehensive description of the care home system viewed through resilient health care theory.

The ability of staff, teams and organisations to adapt and adjust to anticipated and unanticipated variation is vital for sustaining operations and achieving good outcomes.

Together, the descriptions of work as done and themed resilience indicators begin to show how safe, high quality care is created in the context of complex interactions between people and aspects of the socio-technical environment such as the built environment, leadership, and the management of external pressures [46].

Staff adapt to dynamic, variable conditions and coordinate and make priority decisions based on experience in the job. There are restrictions and difficulties in terms of the physical environment and the burden of paperwork for information gathering, monitoring and responding. Care is still relatively fragmented at times but there are concerted efforts to focus holistically on residents’ preferences and quality of life.

Managers and staff on the whole support good outcomes through adapting and adjusting to variable resident needs, environmental conditions and everyday events. There have been calls for investment in the front-line carer workforce, including strengthening training requirements and opportunities, and creating advanced roles [47]. But training can only be part of the approach. System resilience is underpinned not just via care skills and knowledge garnered through training and guidelines, but by non-technical skills (communication, coordination, decision making), making best use of design and physical infrastructure, and by experiential or ‘tacit’ knowledge that is substantially learned ‘on the job’. There is little training provided for key coordination activities, such as prioritising tasks, anticipating, responding to interruptions, and synthesising conflicting information, which are shown to be vital in everyday care delivery.

Paperwork generally acts as a barrier to care, as previously reported [48]. The move to electronic systems in many care homes could lead to more timely and efficient recording of information, and better care through monitoring and the sharing of accurate information. However, the benefits (and potential negative consequences) of this move warrant further investigation with all relevant stakeholders. Attention is turning in recent years towards organizational-level approaches to implementation and improvement [49]. Interventions must be based on models of healthcare systems and theories of how people, tools and resources interact if they are to be consistently effective [50,51].

Interventions need to support providers’ ability to adapt and adjust as they meet the challenges of providing safe, high-quality care for older people. These can be targeted either at reducing misalignments, supporting the potential for organisational resilience, or both, but must be based on studying ‘work-as-done’. The complex and varying needs and preferences of residents means that care homes are dynamic environments that pose many difficult priority decisions for the people working in them (advanced dementia care was described by one inspector as ‘fiendishly difficult’). Focusing on care omissions can be important [52] but this needs to be complemented by identifying and supporting flexible local pathways to success.

### Summary of findings and recommendations

- Work conditions in care homes are inherently dynamic and this needs to be considered a complex, specialised area of care.
- Flexible use of staff, physical space and documentation, based on local conditions, should be supported where a valid case can be made for their effectiveness and where they are based on structured thinking about risks and benefits, involving multiple perspectives.
- Care plans should be simplified and duplication of information removed. Family input is vital. Training on how to produce care plans should be strengthened. Electronic solutions for care monitoring and planning have the potential to improve care via sharing of more complete, accurate and timely information in an efficient way; potential benefits and unintended consequences both need further investigation.
- Task prioritization and mindful adaptation to variable conditions and multiple goals should form part of training. Currently this is tacit knowledge, learned through ‘trial and error’ on the job. Such training might take the form of simulated practice situations or vignettes that mimic real-life scenarios and allow staff to safely work through and discuss them, as is common in hospital care [53].
- Functions such as local care home liaison groups which support multi-disciplinary input (nutritional, psychological, social, medical and dental, physiological etc) to care plans and assessments are recommended.
- Organisational considerations must be incorporated into the design of guidance and implementation efforts, even for seemingly simple activities. We reported (Table 2) how staff feel the physical design and layout of homes affects planning and responding to need. Some level of basic task analysis for key activities involving staff, residents and families is recommended to find the optimal way to achieve good care in the given built environment, and/or how re-design might help.
- As well as assessing care provision holistically there is a need for formal recognition that all risk cannot be eliminated with a vulnerable population, finite resources, and a goal to maintain independence and mobility; inspection should focus on quality of life and risk being as low as reasonably practicable [54].

### Strengths and limitations

Organisational factors are frequently cited as important in care practice [55], yet organizational theories themselves are under-utilized in supporting routine delivery of care. [56,57] The I-SCOPE study applied such theory to the care home setting in a systematic way using a model of Resilient Healthcare, undertaking extensive multi-modality fieldwork across a range of settings and fully involving a wide range of stakeholders. A further strength was the inclusion of residents and family members under principles of process consent [58] after ethical amendment. Residents with dementia are excluded from providing insight and perspective in many studies due to traditional ethical/ consent requirements. Staff were able to engage this vulnerable group, helping residents to share their experiences in a structured but non-threatening way.

This paper reports on qualitative research based on rich description of a small number of self-selecting participant sites where people were motivated to take part. The findings were validated at Workshops with other stakeholders and are worthy of further validation efforts on a wider scale. The study took place prior to the major disruption of the COVID-19 pandemic and a number of changes to everyday work have since been observed. However more recent studies have shown that there is an ongoing need to make difficult trade-off decisions and co-ordinate dynamic responses to local conditions (e.g. Marshall et al. stress the importance of ‘the ability of care home staff to identify and solve emerging issues in care homes’ [59]). There is also recognition of the importance of organisational and workforce management strategies, as well as known drivers such as staffing ratios and access to critical resources [60].

## Conclusions

This study has made recommendations to support the ability of staff, teams and organisations to adapt and adjust to anticipated and unanticipated variation which is vital for sustaining operations and achieving good outcomes. Everyday work in the care home sector is complex and pressured. Systems approaches are vital, and improvement should prioritise design and configuration of work system elements thus ‘making it easy to do the right thing’; there are opportunities for providers and inspectors working together to support adaptive capacity, (through for example task analysis, simulated practice, and design of spaces and work procedures based on ergonomic principles) supporting how to do things under variable conditions, rather than simply telling people what to do based on ideal circumstance [61–63].

The need for good organisational science applied to care home improvement has arguably never been greater. There is now a wealth of literature [64,65] on the difficulty of intervening successfully in healthcare systems, which are complex [66] and involve dynamic interacting components [67]. We understand the notable challenge of finding accessible applications of organisational theory to understand systems into which interventions are designed [68]. Employing such methods is however important for sustainable change [69]. Finally, when seeking to design or implement interventions, realistic models of consent should be explored to include as many residents as possible in giving perspectives on and co-designing care.

## Supporting information

S1 File Consolidated criteria for reporting qualitative studies (COREQ) checklist. (DOC)
